# An unexpected alternative viologen electron mediator site in tungsten‐containing formate dehydrogenase

**DOI:** 10.1002/1873-3468.70373

**Published:** 2026-05-28

**Authors:** Eleni G. Poloniataki, Yong Hwan Kim

**Affiliations:** ^1^ School of Energy and Chemical Engineering, Ulsan National Institute of Science and Technology (UNIST) Republic of Korea; ^2^ Graduate School of Carbon Neutrality, Ulsan National Institute of Science and Technology (UNIST) Republic of Korea

**Keywords:** CO_2_ reduction, electron transfer, ethyl viologen, flavin mononucleotide, formate dehydrogenase

## Abstract

The molecular mechanisms by which artificial electron mediators, such as ethyl viologen (EV), interact with tungsten‐containing formate dehydrogenases (FDHs) during reversible CO_2_ reduction remain poorly understood. Here, we reveal an unexpected alternative mediator interaction site in FDH1 of *Methylorubrum extorquens* AM1. Removing the native flavin mononucleotide cofactor abolishes NAD^+^‐dependent activity but preserves EV‐driven catalysis. Through mutagenesis, kinetic analysis, and molecular docking, we identified a cooperative aromatic network—comprising residues F232, F471, and Y329—that stabilizes EV via stacking interactions near the proximal B1 iron–sulfur cluster. Disrupting these residues impairs EV‐mediated electron transfer without destabilizing global structure. These findings reveal a dual interaction strategy for artificial mediators in tungsten FDHs, offering a structural framework to rationally engineer biocatalysts for CO_2_ conversion.

## Graphical abstract

An unexpected alternative interaction site for ethyl viologen was identified in formate dehydrogenase 1 from *Methylorubrum extorquens*. Combined mutagenesis, kinetic analysis, and docking revealed that aromatic residues near an iron–sulfur cluster enable flavin mononucleotide‐independent electron transfer, offering a framework for engineering improved biocatalysts for carbon dioxide conversion.

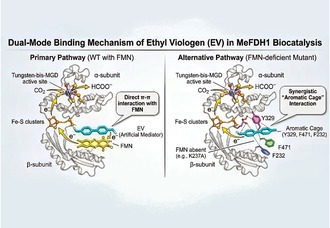

## Abbreviations

**B1**, B1 iron–sulfur cluster

**CD**, circular dichroism

**CO**_**2**_, carbon dioxide

**EV**, ethyl viologen

**EV** ^**+**^, reduced ethyl viologen radical

**EV** ^**2+**^, oxidized ethyl viologen

**FDH**, formate dehydrogenase

**Fe–S**, iron–sulfur

**FMN**, flavin mononucleotide

***Me*FDH1**, formate dehydrogenase 1 from *Methylorubrum extorquens* AM1

**MGD**, molybdopterin guanine dinucleotide

**NAD** ^**+**^, nicotinamide adenine dinucleotide (oxidized)

**NADH**, nicotinamide adenine dinucleotide (reduced)

**WT**, wild type

Anthropogenic greenhouse gas emissions represent a major driver of global climate change, with carbon dioxide (CO_2_), methane (CH_4_), nitrous oxide (N_2_O), and fluorinated gases contributing to atmospheric heat retention and global warming [1]. The resulting climate change is manifested through increasingly frequent extreme weather events, rising sea levels, and widespread disruption of natural ecosystems [2]. Addressing these challenges requires not only reductions in greenhouse gas emissions but also the development of strategies to utilize CO_2_ as a resource.

Industrial activities account for nearly 24% of global CO_2_ emissions, making this sector a key target for mitigation efforts [3, 4]. In parallel with emission reduction, carbon capture and utilization (CCU) has emerged as a complementary approach in which captured CO_2_ is converted into fuels and commodity chemicals, thereby displacing fossil‐derived raw materials and contributing to the decarbonization of hard‐to‐abate sectors [5]. Industrial off‐gases frequently contain not only CO_2_ but also carbon monoxide (CO), nitrogen (N_2_), and hydrogen (H_2_), which are increasingly recognized as abundant feedstocks for sustainable chemical and energy conversion technologies [6, 7, 8, 9, 10, 11]. Direct utilization of such mixed off‐gases (rather than purified single‐component feeds) is attractive because it can integrate with existing industrial infrastructure and has already progressed to commercial‐scale gas fermentation for converting CO/CO_2_/H_2_ streams into fuels and chemicals [12, 13].

Nowadays, biocatalysts are receiving growing attention for their ability to treat and valorize gaseous compounds directly from industrial flue streams, owing to their outstanding performance under mild reaction conditions [10, 14, 15, 16]. These enzymatic systems commonly exhibit high substrate specificity, remarkable tolerance to inhibitors such as sulfur compounds and oxygen, and maintain catalytic activity even in dilute gas mixtures—attributes rarely achieved by conventional catalysts [15, 17, 18]. Moreover, a key advantage of biocatalytic approaches is that enzymatic waste–gas conversion can be performed without the need for prior gas capture or extensive pre‐treatment, thereby simplifying process integration and reducing operational costs [18, 19, 20].

Formate is an attractive product of CO_2_ conversion, as it serves as a stable intermediate in the CO_2_‐to‐methanol and CO_2_‐to‐methane pathways, a valuable chemical feedstock for numerous industrial processes, and a practical liquid energy carrier [21, 22]. Among the available CO_2_‐conversion strategies, biocatalytic reduction to formate by formate dehydrogenase (FDH) is considered particularly promising [23]. FDHs typically contain a metal‐based active site—most commonly incorporating molybdenum or tungsten—that catalyzes the reversible interconversion between formate and CO_2_ [22].

**Fig. 1:**
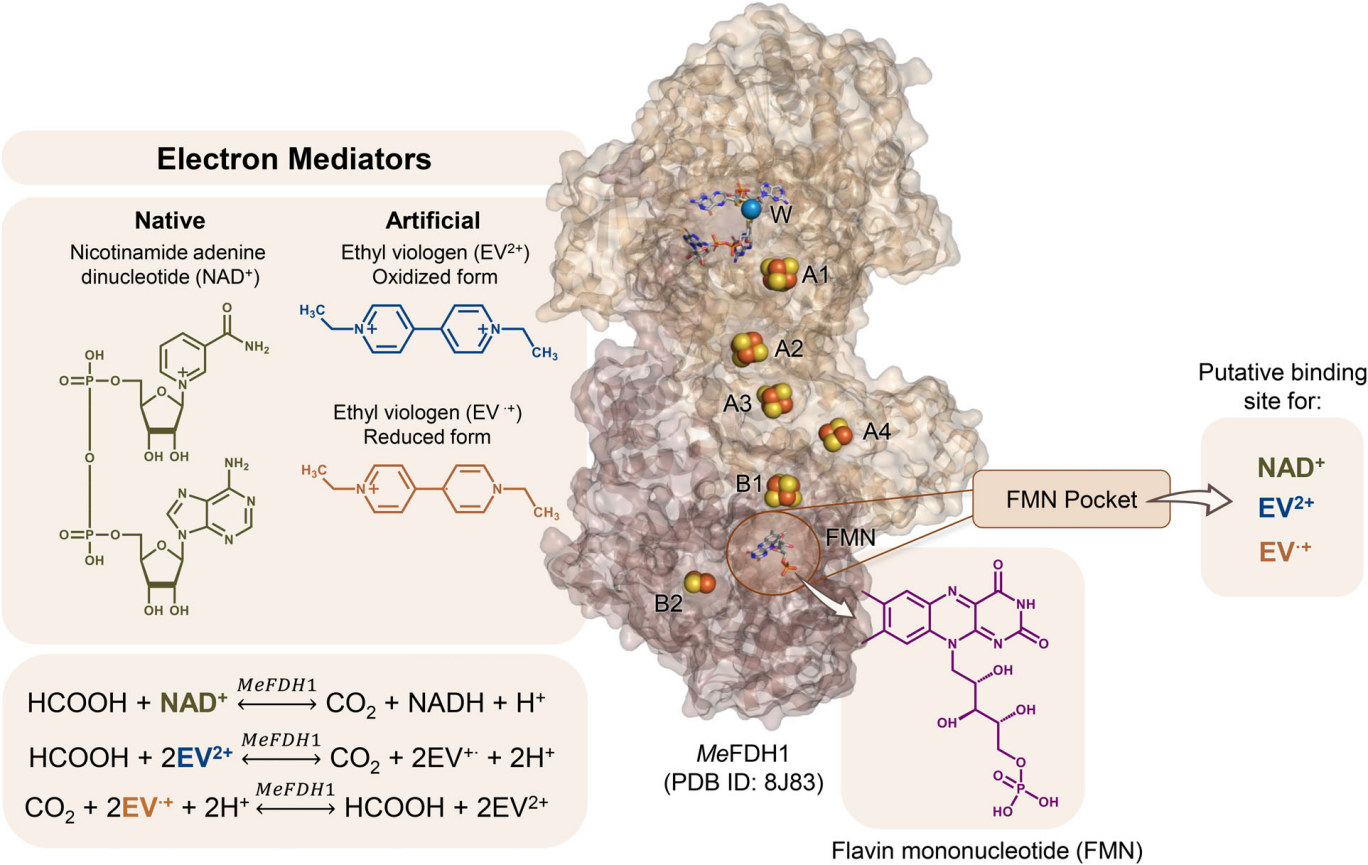
Structure of *Me*FDH1 and reactions catalyzed with various electron mediators. In the upper left, the electron mediators used by *Me*FDH1 are shown, including the native electron mediator nicotinamide adenine dinucleotide (NAD^+^; left) and the artificial mediator ethyl viologen (EV; right), depicted in its oxidized (EV^2+^, upper) and reduced radical (EV^+·^, lower) forms. In the lower left, the corresponding *Me*FDH1‐catalyzed reactions are listed: from top to bottom, formate oxidation with NAD^+^, formate oxidation with oxidized EV, and CO_2_ reduction with reduced EV. At the center, the surface representation of *Me*FDH1 highlights the W‐bis‐MGD active site (W) in the α‐subunit (orange) and the flavin mononucleotide (FMN) cofactor in the β‐subunit (brown), connected by a relay of Fe–S clusters (A1–A4 and B1–B2) mediating long‐range electron transfer. On the right, the chemical structure of FMN is shown. NAD^+^ and EV (EV^2+^/EV^•+^) binding positions are indicated schematically as putative sites in the FMN pocket region (based on the β‐subunit nucleotide‐binding pocket and EV docking), whereas FMN is shown at its resolved location in 8J83.

Formate dehydrogenase 1 from *Methylorubrum extorquens AM1* (*Me*FDH1) functions as an effective biocatalyst for CO_2_ reduction [17, 24]. The enzyme is a heterodimer composed of an α‐subunit containing the tungsten‐based active site and a β‐subunit harboring flavin mononucleotide (FMN), which mediates electron transfer from formate to the native electron mediator nicotinamide adenine dinucleotide (NAD^+^) [25] (Fig. 1). For the enzymatic transformation of CO_2_ into formate, an electron mediator is crucial to facilitate the required redox reactions [6]. Although the nicotinamide adenine dinucleotide is the native electron mediator, its high cost and chemical instability—particularly in the reduced form (NADH)—limit its applicability in large‐scale biocatalytic processes [26, 27].

Viologens have therefore emerged as attractive artificial electron mediators for gas‐converting metalloenzymes, including *Me*FDH1 [28, 29, 30, 31], and have been widely used as artificial redox cofactors in electrochemically driven FDH systems for CO_2_‐to‐formate conversion [32]. Beyond electrochemical platforms, photo‐assisted systems have also leveraged viologen redox mediators and/or photosensitizers to supply reducing equivalents to FDH for CO_2_‐to‐formate conversion, including Ru‐complex–sensitized architectures and semi‐artificial photoelectrochemical devices [33, 34, 35]. Although methyl viologen (MV) is widely used as a cofactor analogue in FDH studies, its well‐established acute toxicity raises safety concerns for large‐scale deployment of viologen‐mediated biocatalytic processes [36, 37]. We therefore selected ethyl viologen (EV) as the mediator in this work as a safer alternative for scale‐up, since EV salts are typically classified with a less severe acute hazard profile than MV in safety documentation [38]. From a thermodynamic standpoint, this substitution remains appropriate because EV preserves the low‐potential redox characteristics of viologen mediators. At neutral pH, the CO_2_/formate redox couple (E°′ ≈ −0.41 V vs SHE) is thermodynamically lower than the NAD^+^/NADH potential (E°′ ≈ −0.32 V), rendering the latter insufficient to drive CO_2_ reduction [18, 39]. By contrast, EV has a redox potential close to that of MV and sufficiently negative (E°′ ≈ −0.45 V) to support electron transfer to *Me*FDH1 for CO_2_ reduction under mild conditions [18] (Fig. 1). Therefore, EV provides a practical compromise between safety and catalytic thermodynamic suitability for mediator‐assisted CO_2_ reduction and compatibility with ambient reaction conditions [18, 40, 41]. Despite their widespread use, however, the fundamental aspects of how artificial electron mediators such as EV interact with metalloenzymes—including the location of their interaction site, their affinity, and the extent to which these interactions influence catalytic activity—remain poorly understood [42].

In this study, we identified the interaction site of the artificial electron mediator EV in *Me*FDH1 through site‐directed mutagenesis, docking simulations, and a kinetic study, including activity and affinity measurements. By systematically examining the FMN and aromatic residues in its vicinity, we pinpointed the critical sites involved in EV interaction during electron transfer in both the formate oxidation and CO_2_ reduction. These analyses further revealed how substitutions at these residues influence the strength of EV interaction, resulting in corresponding changes in *Me*FDH1 catalytic activity. Mapping the EV interaction site in *Me*FDH1 will enable rational engineering of the mediator‐interacting pocket to enhance electron‐transfer efficiency and reduce the required EV concentration for CO_2_ reduction. This structural insight will also support the design of electrode‐coupled or polymer‐linked biocatalytic systems, advancing *Me*FDH1 toward efficient and sustainable CO_2_‐to‐formate conversion under mild conditions.

## Materials and methods

### Cloning, protein expression, and purification

Unless stated otherwise, all chemical reagents used for culture, protein purification, and enzymatic assays were purchased from Sigma‐Aldrich (St. Louis, MO, USA) and used without further purification. The pCM110 expression vector, containing the *PmxaF* promoter, a pET RBS (ribosomal binding site), a tetracycline resistance gene, and the *fdh1α* and *fdh1β* genes (GenBank accession No. ACS42636.1 (α‐subunit), ACS42635.1 (β‐subunit)), was used for expression of *Me*FDH1. A hexahistidine tag (His6 tag) was fused to the C‐terminus of the α‐subunit. Site‐directed mutagenesis was performed to generate *Me*FDH1 variants. Following PCR amplification, DpnI (0.5 U·μL^−1^) was added, and the reaction was incubated at 37 °C for 30 min to digest the parental plasmid. The reaction mixture was transformed into *Escherichia coli* DH5α, and plasmids were isolated and verified by DNA sequencing (Macrogen, Inc., Seoul, Republic of Korea).

Sequence‐verified plasmids were transformed into *Methylorubrum extorquens* AM1 (ATCC 14781; GenBank accession CP001510.1) lacking the native *fdh1* and *kan* genes, as described previously [24], by electroporation using a Bio‐Rad MicroPulser (Bio‐Rad Laboratories, Inc., Hercules, CA, USA) at 2.5 kV with a  0.2 cm electroporation cuvette. Transformants were selected on medium containing rifamycin and tetracycline. Details of plasmids and primers used in this study are provided in Table S1.

*Methylorubrum extorquens* AM1 was cultivated in modified medium [24] supplemented with 4 g·L^−1^ succinate and 30 μm sodium tungstate. Antibiotics were added at final concentrations of 50 μg·mL^−1^ rifamycin and 10 μg·mL^−1^ tetracycline. Cultures were incubated aerobically at 30 °C with shaking at 170 rpm in 1 L baffled Erlenmeyer flasks (200 mL working volume). After 24 h, methanol was added to a final concentration of 0.5% (v/v) to induce expression. Cells were harvested 24 h after induction by centrifugation at 7000× ***g*** for 10 min at 4 °C and stored at −70 °C until use.

All purification steps were carried out under anaerobic conditions in an anaerobic chamber (Coy Laboratory Products, Inc., Grass Lake, MI, USA) under an atmosphere supplied by an N_2_/H_2_ gas mixture (3.9% H_2_ balanced with N_2_), with chamber H_2_ maintained at ≥ 2%. Cell pellets were resuspended and disrupted by sonication (20 min total, 2 s pulses, 40% amplitude) in buffer containing 50 mm MOPS, 200 mm NaCl, and 20 mm imidazole, and pH 7.0. Cell debris was removed by centrifugation at 11 000× ***g*** for 20 min at 4 °C. The supernatant was loaded onto a 1 mL Ni‐NTA agarose column (QIAGEN, Hilden, Germany) equilibrated with the same buffer. After washing, bound protein was eluted with buffer containing 50 mm MOPS, 200 mm NaCl, and 300 mm imidazole, pH 7.0. Protein concentrations were determined by absorbance at 280 nm using a NanoDrop One spectrophotometer (Thermo Fisher Scientific, Waltham, MA, USA). The extinction coefficient of *Me*FDH1 (*ε* = 8.89 L·g^−1^·cm^−1^ for a 1% solution) was calculated using EMBOSS Pepstats. Unless otherwise stated, Ni–NTA eluates were used for enzymatic activity and kinetic assays to preserve non‐covalently associated FMN. FPLC polishing was performed only for FMN occupancy comparisons and FMN add‐back experiments (Tables S2 and S3).

### FPLC polishing (size‐exclusion chromatography)

Ni–NTA eluates were further purified by size‐exclusion chromatography (SEC) using a HiLoad 16/600 Superdex 200 pg column (Cytiva, Marlborough, MA, USA) operated inside an anaerobic chamber. The column was equilibrated and run in 50 mm MOPS, 200 mm NaCl (pH 7.0) at a flow rate of 1.0 mL·min^−1^. Protein was loaded using a 2 mL sample loop, and elution was monitored by UV absorbance at 280 nm. *Me*FDH1 eluted at a retention volume of 68.432 mL; fractions corresponding to the main peak were pooled and used for FMN occupancy measurements and FMN add‐back experiments (Tables S2 and S3).

### Enzyme activity and kinetic analysis

*Me*FDH1 activity was determined by monitoring formate oxidation using either NAD^+^ or EV as an electron mediator, as well as CO_2_ reduction using reduced EV. Formate oxidation assays were performed in 200 mm potassium phosphate buffer (pH 6.3) containing 30 mm potassium formate and either 0.5 mm NAD^+^ (NAD^+^, *ε*_340_ = 6220 M^−1^·cm^−1^) or 10 mm oxidized ethyl viologen (EV^2+^). EV^2+^ reduction to the viologen radical (EV^•+^) was monitored at 578 nm (EV^•+^, *ε*_578_ = 10 000 M^−1^·cm^−1^).

CO_2_ reduction activity was measured by following the oxidation of reduced ethyl viologen radical (EV^•+^) at 578 nm (EV^•+^, *ε*_578_ = 10 000 M^−1^·cm^−1^) in 200 mm potassium phosphate buffer (pH 6.3) containing 100 mm potassium bicarbonate as the carbon dioxide source and 0.12 mm reduced EV^•+^. EV was reduced by incubation with zinc metal at a tenfold mole excess, and residual zinc was removed by filtration through a 0.2 μm syringe filter. All assays were performed anaerobically at 30 °C and measured in triplicate using a Cary 60 UV/visible spectrophotometer (Agilent Technologies, Inc., Santa Clara, CA, USA).

All enzyme assays were prepared and performed inside anaerobic chambers (Coy Laboratory Products) maintained at ≥ 2% H_2_ balanced with N_2_. Oxygen levels were monitored continuously using CAM‐12 H_2_/O_2_ detectors (Coy Laboratory Products), and the chamber H_2_ concentration was maintained at ≥ 2%. All buffers were autoclaved, transferred hot into the chamber, and equilibrated overnight prior to use to minimize dissolved O_2_. Consumables (tubes, tips, cuvettes) and equipment components were kept inside the chamber overnight before experiments. UV/Vis measurements were carried out using a Cary 60 spectrophotometer installed inside the anaerobic chamber (Fig. S1).

Relative activity values (%) were calculated by dividing the specific activity of each mutant by that of the matched wild‐type Ni–NTA preparation measured in parallel under the same assay conditions and multiplying by 100. This approach enables controlled comparison across variants prepared under the same expression and purification workflow and does not imply normalization to a fully FMN‐occupied holoenzyme state. Reproducibility of wild‐type specific activities across independent Ni–NTA purifications is reported separately in Table S4.

The kinetic parameters for formate oxidation using EV^2+^ were determined under the same buffer and temperature conditions by varying the concentration of oxidized ethyl viologen (EV^2+^) at a fixed formate concentration (30 mm). For wild‐type and single mutants, EV^2+^ was varied at 0.3125, 0.625, 1.25, 2.5, and 5 mm. For the double, triple, and quadruple mutants, an additional EV^2+^ concentration point at 10 mm was included to improve parameter estimation. Data were collected in triplicate and analyzed to derive *k*_cat_ and *K*_M_ values. Errors for catalytic efficiencies (*k*_cat_/*K*_M_) were propagated from the standard deviations of *k*_cat_ and *K*_M_ using standard error propagation for ratios.

### FMN quantification by HPLC

FMN content was quantified by using an Agilent 1200 system equipped with a diode‐array detector and a Prep‐C18 column (250 × 4.6 mm). The column temperature was maintained at 30 °C, and detection was performed at 264 nm. Solvent A consisted of 5 mm ammonium acetate, and solvent B was methanol (HPLC grade). Solvents were filtered through 0.22 μm membranes and degassed by ultrasonication.

The column was equilibrated at a flow rate of 0.8 mL·min^−1^ with 85% solvent A and 15% solvent B. After sample injection, a linear gradient from 15% to 37.5% solvent B over 8 min and from 37.5% to 50% over 5 min was applied, followed by isocratic elution at 50% solvent B for 5 min, and then lowered to 15% in 5 min. FMN eluted at approximately 11 min. Calibration curves were generated using 50 μL injections of FMN standards (40–800 pmol) prepared in 50 mm MOPS, 100 mm NaCl, pH 7.0.

Protein samples (5–20 μm) in the same buffer were heated at 100 °C for 10 min to release bound FMN, centrifuged to remove precipitated protein, and filtered prior to injection. All sample preparation and measurements were performed under reduced light conditions.

### FMN occupancy measurements and FMN add‐back experiments

FMN occupancy was calculated as (mol FMN/mol *Me*FDH1) × 100. For Table S2, FMN occupancy was measured for samples collected after Ni–NTA elution and after FPLC polishing from three independent purifications; each sample was quantified in triplicate. For Table S3, FPLC‐purified *Me*FDH1 was incubated with exogenous FMN at 1×, 2×, 5×, or 10× molar equivalents (relative to *Me*FDH1) for 2 h at room temperature inside an anaerobic chamber and protected from light. Following incubation, free FMN was removed by buffer exchange using Vivaspin centrifugal filters (100 kDa MWCO) (Sartorius Stedim Lab Ltd, Stonehouse, Gloucestershire, UK): samples were centrifuged at 12 300 ***g*** for 10 min and washed four times with 50 mm MOPS, 100 mm NaCl (pH 7.0). FMN content was measured in the recovered protein fraction. Following FMN quantification, specific activities were measured for formate oxidation with NAD^+^, formate oxidation with EV^2+^, and CO_2_ reduction with EV^•+^ under the standard assay conditions described above.

### Docking simulation

Molecular docking was performed to examine potential interaction modes between *Me*FDH1 and EV. The *Me*FDH1 structure (PDB ID: 8J83) was obtained from the Protein Data Bank and prepared by removing water molecules and optimizing the structure as required. Docking calculations were carried out using CB‐Dock2 [43, 44], which identifies potential binding cavities and performs docking using AutoDock Vina. Docking poses were visualized and analyzed using pymol (version 2.5.1). Interaction energies were calculated using discovery studio (Dassault Systèmes, Vélizy‐Villacoublay, France).

### Circular dichroism spectroscopy

For circular dichroism spectroscopy, purified proteins were exchanged into 50 mm sodium phosphate buffer, pH 7.0, using Vivaspin centrifugal filters (100 kDa molecular weight cutoff). Measurements were performed at a protein concentration of 0.2 mg·mL^−1^ using a sample volume of 300 μL. Far‐UV circular dichroism spectra were recorded from 190 to 260 nm at 25 °C using a Jasco J‐1500 circular dichroism spectropolarimeter, with a scanning speed of 50 nm·min^−1^. Each spectrum represents the average of five accumulated scans. Secondary structure content was estimated from the recorded spectra using the bestsel server.

## Results and discussion

### Role of FMN in viologen‐mediated electron transfer by *Me*FDH1

Figure 1 summarizes the structural and redox framework of *Me*FDH1 and the electron mediators examined in this study. *Me*FDH1 is a heterodimeric enzyme composed of an α‐subunit harboring the tungsten–bis‐molybdopterin guanine dinucleotide (W‐bis‐MGD) active site and a β‐subunit containing a FMN cofactor, with the two subunits connected by a relay of Fe–S clusters (A1–A4 and B1–B2) that enable long‐range electron transfer [25]. As illustrated in the reaction schemes, *Me*FDH1 catalyzes either formate oxidation or CO_2_ reduction depending on the redox state of the electron mediator [18, 45]. The native cofactor NAD^+^/NADH supports only formate oxidation, and FMN is well established as the primary interaction site for NAD(H), receiving electrons generated during formate oxidation [42]. In contrast, the artificial mediator EV, in both oxidized and reduced states, enables reversible interconversion of CO_2_ and formate under mild conditions [18, 45].

**Fig. 2:**
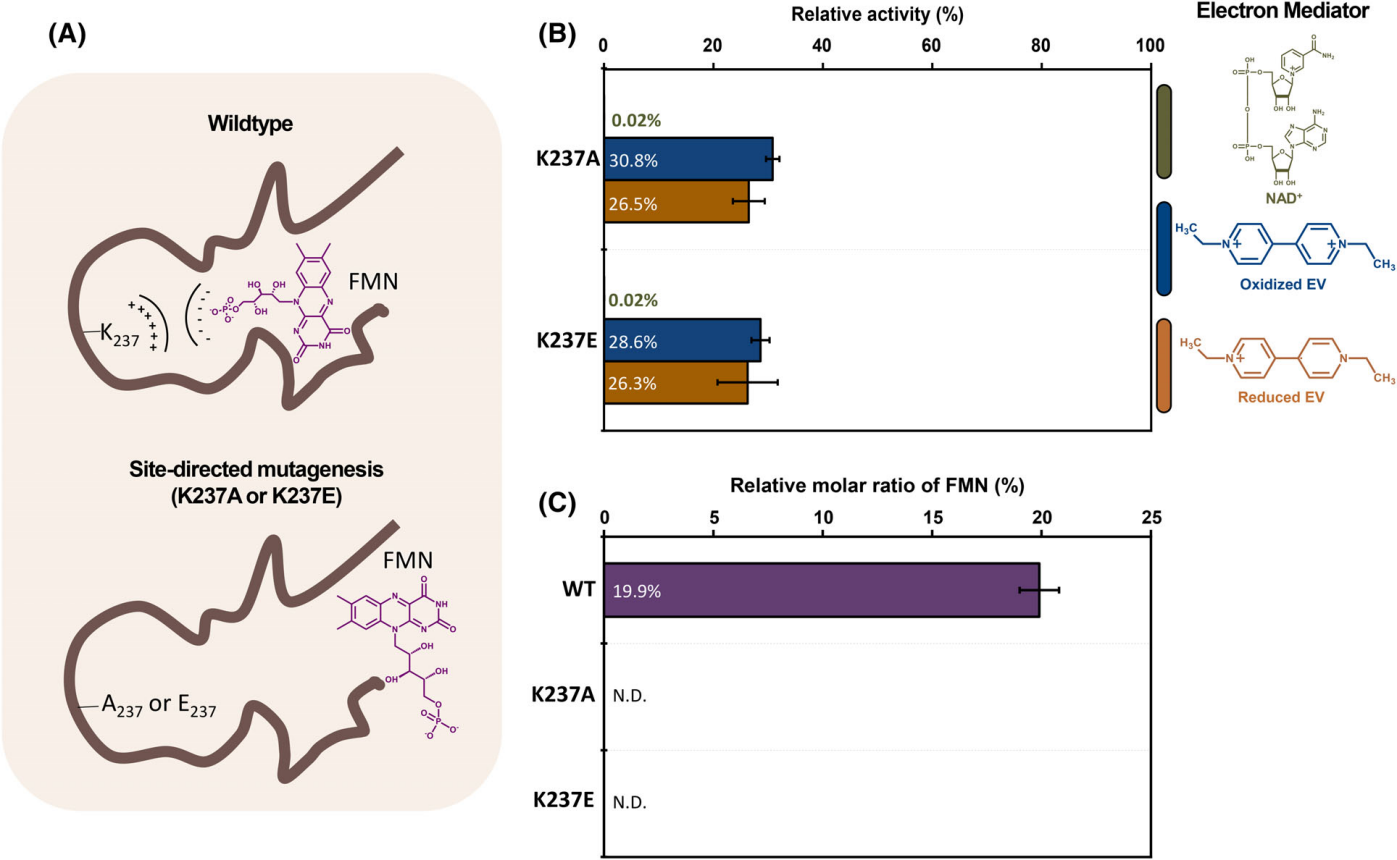
Impact of residue K237 on FMN association and *Me*FDH1 activity. (A) Schematic representation of the FMN‐binding environment in wild‐type *Me*FDH1 (top) and the K237A and K237E variants generated by site‐directed mutagenesis (bottom). In the wild‐type enzyme, the positively charged residue K237 stabilizes FMN through electrostatic interactions with its phosphate group, whereas substitution with alanine or glutamate disrupts this interaction, leading to loss of FMN association. (B) Relative activities of wild‐type *Me*FDH1, K237A, and K237E variants with NAD^+^, oxidized ethyl viologen (EV^2+^), and reduced ethyl viologen (EV^+·^) as electron mediators. Relative activities were calculated by dividing the activity of each variant by that of the matched wild‐type Ni–NTA preparation obtained under the same purification conditions and multiplying by 100. Both K237A and K237E show almost complete loss of NAD^+^‐dependent activity and reduced EV‐dependent activity. (C) Relative molar ratio of FMN content in purified *Me*FDH1 and its variants. The wild‐type value shown here (19.9%) corresponds to the representative Ni–NTA preparation quantified in parallel with the variant samples in this experiment. Across independent wild‐type purifications, Ni–NTA preparations exhibit substoichiometric FMN occupancy (23.7 ± 8.4%; Table S2). FMN was not detected (N.D.) in K237A or K237E under the same quantification method, indicating that substitution at residue 237 strongly disrupts FMN association. Error bars in panels (B, C) represent standard deviation (SD) from three measurements (*n* = 3).

Despite the widespread use of the *Me*FDH1–EV system in biocatalysis, the molecular basis of this interaction has not been previously characterized [18, 46]. To investigate whether FMN constitutes the principal interaction region for EV, residue K237 in the β‐subunit was substituted with alanine or glutamic acid. Lysine 237 contributes to the stabilization of the FMN phosphate group through electrostatic interactions within the flavin‐binding pocket (Fig. 2A). To promote FMN dissociation, substitution with alanine was designed to remove this electrostatic interaction, whereas substitution with glutamic acid was expected to introduce electrostatic repulsion, thereby weakening FMN association.

The effects of the K237 substitutions on *Me*FDH1 activity are shown in Fig. 2B, with activities expressed relative to the wild‐type enzyme. For each variant, formate oxidation with NAD^+^, formate oxidation with oxidized EV, and CO_2_ reduction with reduced EV were examined. As expected, the NAD^+^‐dependent activity was almost completely abolished in both K237A and K237E variants (0.02%), consistent with the absence of FMN confirmed by high‐performance liquid chromatography (HPLC) (Fig. 2C). The representative wild‐type preparation shown in Fig. 2C (19.9% occupancy) falls within the range observed across independent purifications (Table S2). In contrast, both EV‐dependent reactions retained substantial residual activity. Specifically, K237A retained 30.8% activity for formate oxidation and 26.5% for CO_2_ reduction, while K237E retained 28.6% and 26.3%, respectively. These relative activities were calculated by dividing the specific activity of each variant by that of the matched wild‐type Ni–NTA preparation measured in parallel under the same purification conditions and multiplying by 100. Thus, they represent comparative performance within a consistent preparation state, rather than normalization to a fully FMN‐occupied holoenzyme. This comparison reflects the behavior of a preparation containing a mixed population of FMN‐bound and FMN‐depleted enzyme states, arising from partial loss of non‐covalently bound FMN during purification. As both substitutions produced comparable effects on FMN dissociation and residual activity, K237A was selected for subsequent mutational analyses. Notably, these results indicate that *Me*FDH1 retains a flavin‐independent, ethyl viologen–supported electron‐transfer capability.

Quantification of FMN content revealed that FMN occupancy in our *Me*FDH1 preparations was consistently substoichiometric and dependent on purification extent. Across three independent purifications performed under anaerobic conditions, Ni–NTA eluates showed an average FMN occupancy of 23.7 ± 8.4%, whereas additional SEC‐FPLC polishing reduced occupancy to 6.7 ± 1.4% (Table S2), consistent with loss of non‐covalently associated FMN during further purification. Because FMN is non‐covalently bound and readily lost during extended purification, Ni–NTA eluates were used for activity assays unless otherwise noted, and FMN occupancy is reported as an empirical property of the preparation under the specified workflow (Tables S2 and S3).

We attempted to restore FMN loading by incubating FPLC‐purified *Me*FDH1 with exogenous FMN (1–10 molar equivalents) under anaerobic and dark conditions followed by removal of free FMN; however, FMN occupancy remained low (~ 7–10%) and did not increase substantially (Table S3). Similar limitations have been reported for *Me*FDH1 [47, 48], suggesting that cofactor incorporation and retention are limiting features of this system and motivating future engineering of the FMN pocket environment to enhance FMN binding and stabilize the holo state. Accordingly, relative activities are reported versus the matched wild‐type Ni–NTA preparation.

Consistent with this controlled workflow, specific activities of Ni–NTA purified wild‐type *Me*FDH1 were reproducible across independent purifications (Table S4), supporting that the comparative activity trends reported for variants are not driven by batch‐to‐batch variability. Tables S2, S3 address FMN occupancy and add‐back behavior, whereas Table S4 reports activity reproducibility for Ni–NTA preparations used as the reference state for variant comparisons.

### Docking simulations for *Me*FDH1 with and without FMN

**Fig. 3:**
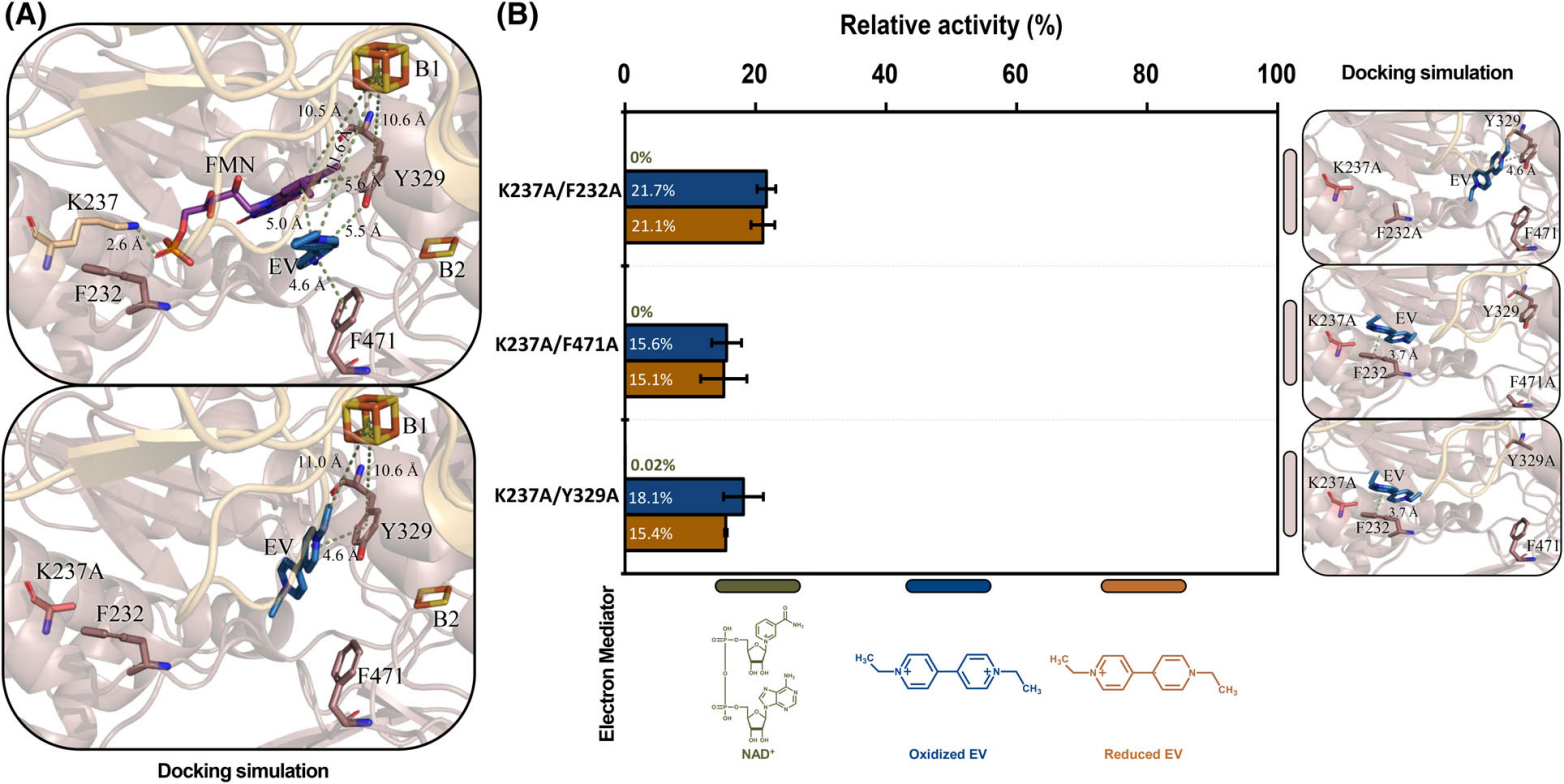
Contribution of aromatic residues near the FMN site to ethyl viologen interaction and *Me*FDH1 activity. (A) Docking simulations showing representative predicted interaction modes of ethyl viologen (EV; blue) in the FMN pocket region of wild‐type *Me*FDH1 (top) and the K237A variant (bottom). The views are oriented to display EV relative to both FMN (purple; wild type only) and the proximal β‐subunit B1 [4Fe–4S] cluster (B1). In the wild‐type enzyme, EV is positioned near the FMN isoalloxazine ring and in proximity to aromatic residues in the FMN pocket environment (including Y329 and F471; brown), consistent with π–π interactions. In the K237A variant, which lacks detectable FMN, the predicted EV pose shifts within the same region. Representative distances are indicated. (B) Relative activities (left) of the K237A/F232A, K237A/F471A, and K237A/Y329A variants compared with wild‐type *Me*FDH1 using NAD^+^, EV^2+^, or EV^+·^ as electron mediators. Relative activity values (%) were calculated by dividing the specific activity of each variant by that of the matched wild‐type Ni–NTA preparation measured in parallel under the same conditions and multiplying by 100. Error bars represent standard deviation (SD) from three measurements (*n* = 3). Docking snapshots (right) show representative EV interaction poses for each variant, illustrating altered π‐stacking interactions upon substitution of F232, F471, or Y329.

**Fig. 4:**
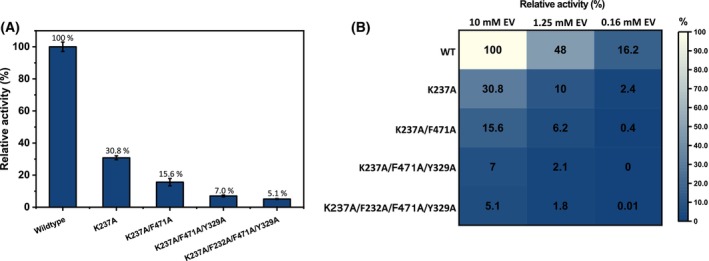
Effects of aromatic‐residue substitutions on EV‐dependent *Me*FDH1 activity. (A) Relative activities of wild‐type *Me*FDH1 and selected single, double, triple, and quadruple variants in the formate oxidation reaction using oxidized ethyl viologen (EV^2+^) as the electron mediator. Relative activities were calculated by dividing the activity of each variant by that of the matched wild‐type Ni–NTA preparation obtained under the same purification conditions and multiplying by 100. Progressive substitution of aromatic residues near the FMN site (F232, F471, and Y329), in combination with K237A, results in an overall decrease in catalytic activity. Error bars represent standard deviation (SD) from three measurements (*n* = 3). (B) Heat map showing relative *Me*FDH1 activities for formate oxidation at three EV concentrations (10, 1.25, and 0.16 mm). Each cell represents relative activity calculated by dividing the activity of each variant by that of the matched wild‐type Ni–NTA preparation measured under the same conditions and multiplying by 100. Lower EV concentrations accentuate activity losses caused by aromatic‐residue substitutions, indicating the importance of these residues for efficient EV‐mediated electron transfer.

Because EV does not have a resolved binding pose in the *Me*FDH1 structure, we use docking to propose plausible EV poses; however, the primary experimental support for the EV interaction region comes from the residue‐specific activity losses across the alanine scan (Table S5), the K237A‐based double/triple/quad series (Figs 3, 4 and Tables S6, S7), and the EV^2+^ concentration‐series kinetics (Table 1), which together demonstrate that residues surrounding the FMN pocket govern productive mediator‐dependent turnover.

**Table 1:** Kinetic parameters of wild‐type *Me*FDH1 and mutant variants in the EV‐dependent formate oxidation reaction. Values represent the Michaelis–Menten constants *K*_M_, turnover numbers *k*_cat_, and catalytic efficiencies *k*_cat_/*K*_M_ for wild‐type *Me*FDH1 and single, double, triple, and quadruple mutants, which were obtained by varying the concentration of oxidized ethyl viologen (EV^2+^). All measurements were performed under identical conditions, and values are reported as mean ± SD (*n* = 3).

Mutation	*K*_M_ (mm)	*k*_cat_ (s^−1^)	*k*_cat_/*K*_M_ (s^−1^·mm ^−1^)
Wild type	0.91 ± 0.004	715.03 ± 17.69	785.76 ± 19.74
K237A	1.16 ± 0.005	179.75 ± 9.31	154.96 ± 8.05
K237E	1.07 ± 0.005	162.83 ± 8.66	152.18 ± 8.11
K237A/F232A	1.74 ± 0.001	202.33 ± 6.42	116.28 ± 3.69
K237A/F471A	1.97 ± 0.009	136.05 ± 11.87	69.09 ± 6.03
K237A/Y329A	2.78 ± 0.0003	95.50 ± 1.91	34.35 ± 0.69
K237A/F471A/Y329A	3.28 ± 0.03	72.80 ± 6.62	22.20 ± 2.03
K237A/F232A/F471A/Y329A	3.13 ± 0.009	69.01 ± 5.68	22.05 ± 1.82

Because substantial EV‐dependent activity persisted after FMN removal, we hypothesized the presence of an alternative EV interaction region. To provide a structural rationale for the experimentally observed residue dependencies, docking simulations were performed for both wild‐type *Me*FDH1 and the flavin‐deficient K237A variant (Fig. 3A). In the wild‐type enzyme, EV was positioned above the FMN isoalloxazine ring, forming a parallel π–π interaction at a distance of approximately 5.0 Å. Additionally, residue F471 formed a second parallel π–π interaction with ethyl viologen at a distance of 4.6 Å, resulting in a sandwich‐like π–π aromatic stacking arrangement involving FMN–EV–F471. Consistent with this, Fig. 3A visualizes the representative EV pose in the FMN pocket region together with the proximal B1 [4Fe–4S] cluster to emphasize the spatial relationship relevant to electron transfer.

Residue Y329 also contributed to this interaction network, forming a vertical π–π interaction with FMN at a distance of 5.6 Å and interacting with EV through its phenolic group at a distance of 5.5 Å. Overall, these interactions suggest that ethyl viologen is stabilized by a combination of flavin‐ and residue‐mediated aromatic contacts in the wild‐type enzyme.

In the K237A variant lacking FMN, EV adopted an alternative binding pose, engaging in a strong parallel π–π interaction with Y329 at a distance of approximately 4.6 Å, while the interaction with F471 was substantially weakened (9.1 Å). Notably, Y329 was positioned within 10.6 Å of the B1 Fe‐S cluster, a distance within the range generally compatible with efficient biological electron tunneling, consistent with a potential role in mediating electron transfer between EV and the metal cluster [49, 50]. A similar interaction pattern was observed for the K237E variant (Fig. S2), further supporting the importance of Y329 in flavin‐independent ethyl viologen interaction.

### Alanine scanning of aromatic residues proximal to FMN

Aromatic amino acids are known to facilitate electron transfer through their delocalized π‐electron systems [30]. Moreover, these residues can engage in direct π–π interactions with EV, making them strong candidates for mediating EV interaction in *Me*FDH1. To guide selection of residues for further analysis, we first performed alanine scanning mutagenesis of aromatic residues positioned around the FMN pocket (within ~ 10 Å of FMN in 8J83) and measured specific activities for NAD^+^‐dependent formate oxidation, EV^2+^‐dependent formate oxidation, and EV^•+^‐dependent CO_2_ reduction. The full screening dataset is provided in Table S5. From this scan, residues showing the most pronounced decreases in EV‐dependent activities were prioritized for subsequent combinatorial analysis. Based on these results, we selected F232, F471, and Y329 for introduction into the K237A background to decouple effects on EV‐dependent catalysis from FMN binding, because K237A eliminates detectable FMN association in our preparations. Notably, the alanine scan also revealed variants with divergent effects on the two electron‐acceptor pathways—for example, F462A retained near–wild‐type EV‐dependent activity while exhibiting strongly reduced NAD^+^‐dependent activity—supporting that NAD^+^‐coupled turnover and viologen‐mediated turnover can be at least partially decoupled in *Me*FDH1.

Figure 3B summarizes the relative activity values (%), calculated by dividing the specific activity of each variant by that of the matched wild‐type Ni–NTA preparation, measured in parallel under the same conditions, and multiplying by 100. As all variants lack FMN, NAD^+^–dependent activity was abolished entirely. For formate oxidation with oxidized EV, relative activities decreased to 21.7% for K237A/F232A, 15.6% for K237A/F471A, and 18.1% for K237A/Y329A. A similar trend was observed for CO_2_ reduction with reduced EV, with relative activities of 21.1, 15.1, and 15.4%, respectively. Among these variants, K237A/F471A consistently showed the lowest activity, suggesting an important role for F471 in organizing ethyl viologen interactions. However, a residual activity of approximately 15% was retained, indicating that EV‐dependent electron transfer in *Me*FDH1 is more complex than can be accounted for by a single interaction and warrants further investigation.

Docking simulations provided a structural rationale for these observations (Fig. 3B, left). In K237A/F232A, EV retained a binding pose similar to that observed for the K237A single mutant, interacting primarily with Y329. In contrast, in K237A/F471A and K237A/Y329A, EV preferentially interacted with F232, forming a strong parallel π–π interaction at approximately 3.7 Å. Although F471 is positioned at a distance of ~9.1 Å from EV in the K237A/F232A mutant, its substitution disrupted EV stabilization at Y329, suggesting that F471 plays an indirect but important role in organizing the local interaction environment. In the absence of F471, EV preferentially associates with F232, placing the mediator in a geometry that is compatible with electron exchange with the nearby B1 Fe–S cluster, given established distance/rate relationships for biological electron tunneling [51]. Collectively, these findings indicate that residues F232, F471, and Y329 function cooperatively to stabilize EV and support electron transfer between the mediator and the B1 iron–sulfur cluster.

### Cumulative effects of multiple aromatic‐residue substitutions

To further assess the cooperative roles of F232, F471, and Y329 in EV–mediated electron transfer, triple and quadruple mutations were constructed. The K237A/F471A/Y329A variant was selected as the triple mutant because the K237A/F471A and K237A/Y329A double mutants exhibited the lowest relative activities among the double‐mutant series. To eliminate all aromatic residues previously implicated in EV interaction, the quadruple mutant K237A/F232A/F471A/Y329A was generated.

Figure 4A shows the relative activities for formate oxidation using oxidized ethyl viologen (EV^2+^) for *Me*FDH1 variants containing single, double, triple, and quadruple mutations, measured at a fixed EV^2+^ concentration (10 mm). Here, the wild‐type value denotes the activity of the matched Ni–NTA purified wild‐type preparation measured in parallel and used as the reference for relative activity calculations. Overall, introducing additional substitutions reduces activity under this assay condition, indicating that residues in the FMN pocket environment collectively influence EV‐dependent turnover. This pattern is consistent with FMN serving as the dominant redox hub under wild‐type conditions, while residues surrounding the FMN pocket modulate EV‐dependent turnover efficiency. Importantly, because Fig. 4A reports single‐point activity at 10 mm EV^2+^, it reflects turnover at that concentration and is not directly equivalent to catalytic efficiency (*k*_cat_ */K*_M_) derived from EV^2+^ concentration series (Table 1). Consistent with this, steady‐state kinetics show that the triple and quadruple mutants display comparable *k*_cat_ */K*_M_ values, despite differences in single‐point activities.

The complete set of specific activities for all mutants and reactions examined in this study is provided in Table S6. Although residual activities of multiple mutants are small relative to wild type, they are mechanistically informative because they show that EV‐dependent catalysis is not completely abolished even when key FMN pocket interactions are disrupted. These low activities act as a sensitive probe of the structural requirements for productive mediator coupling and guide future engineering of the electron‐transfer interface.

Notably, even the quadruple mutant retained a small residual activity of 5.1%. This remaining activity is most likely attributable to the high EV concentration used in this assay (10 mm), which may permit limited electron transfer even in the absence of stabilizing aromatic interactions.

To further evaluate this effect, formate oxidation activities were measured at three different EV concentrations (10, 1.25, and 0.16 mm) for the wild‐type enzyme and selected mutant variants (Fig. 4B). In all cases, activity decreased with decreasing EV concentration. At 0.16 mm EV, the relative activity of the K237A/F471A double mutant was nearly abolished, and activity was completely lost in the triple mutant. The corresponding specific activities and formate oxidation rates at different EV concentrations are summarized in Table S7.

To ensure that the observed loss of specific activity arises from disruption of key amino acid residues involved in the electron‐transfer mechanism rather than from global structural destabilization of the enzyme, the secondary structures of wild‐type *Me*FDH1 and all mutant variants were examined by circular dichroism (CD) spectroscopy. Far‐UV CD spectra (190–260 nm) were recorded for the wild type and single, double, triple, and quadruple mutants to assess potential changes in overall folding. As shown in Fig. S3, all mutants display CD spectra highly similar to that of the wild‐type enzyme, characterized by comparable spectral shapes and intensities. Consistent with this observation, quantitative secondary structure analysis using the BeStSel algorithm (Table S8) revealed no major differences in α‐helical, β‐sheet, or unordered content among the variants. These results demonstrate that the introduced mutations do not cause significant global structural collapse of *Me*FDH1, supporting the conclusion that the reductions in catalytic activity originate from local perturbations of electron‐transfer interactions rather than from loss of overall protein integrity.

### Kinetic impact of FMN removal and aromatic‐residue mutations on EV‐mediated formate oxidation

Steady‐state kinetic parameters for wild‐type *Me*FDH1 and mutant variants in the formate oxidation reaction using ethyl viologen are summarized in Table 1. The wild‐type enzyme exhibited a low *K*_M_ value (0.91 mm) and a high turnover number (*k*_cat_ = 715.03 s^−1^), resulting in a catalytic efficiency (*k*_cat_/*K*_M_) of 785.76 s^−1^·mm ^−1^. In contrast, removal of FMN through the K237A or K237E substitutions led to a pronounced reduction in *k*_cat_ (to approximately 160–180 s^−1^) and an approximately fivefold decrease in catalytic efficiency, while substrate affinity was only modestly affected. These results indicate that FMN plays a dominant role in facilitating efficient electron transfer, as well as in substrate interaction.

Introduction of additional alanine substitutions at aromatic residues near the FMN pocket further decreased EV‐dependent catalytic performance. The K237A/F232A, K237A/F471A, and K237A/Y329A mutants showed increased apparent *K*_M_ values and reduced *k*_cat_ values relative to K237A, resulting in stepwise decreases in catalytic efficiency (*k*_cat_/*K*_M_) (Table 1). Although the K237A/F232A double mutant shows a modest increase in *k*_cat_ relative to K237A, this is accompanied by an increased *K*_M_, resulting in a net decrease in catalytic efficiency (*k*_cat_/*K*_M_) (Table 1). Notably, the triple mutant K237A/F471A/Y329A and the quadruple mutant K237A/F232A/F471A/Y329A exhibited similar catalytic efficiencies (*k*_cat_/*K*_M_ ≈ 22 s^−1^·mm ^−1^), indicating that the additional F232A substitution does not further reduce catalytic efficiency beyond the triple mutant under the conditions used for kinetic analysis (Table 1). Because single‐point activity at 10 mm EV (Fig. 4A) reflects turnover at a fixed mediator concentration (10 mm), whereas *k*_cat_/*K*_M_ reflects catalytic efficiency in the low‐to‐intermediate EV regime; therefore, the two readouts are complementary but not identical. Collectively, these results support that EV‐mediated catalysis can proceed in the absence of FMN, but optimal turnover is sensitive to residues shaping the FMN pocket environment.

To test the model implication that loss of activity arises primarily from disruption of FMN binding rather than from changes in the aromatic environment, we constructed the aromatic triple mutant without altering the FMN‐binding site (F232A/F471A/Y329A). Contrary to the prediction that this variant would retain near–wild‐type activity, the triple mutant retained measurable FMN occupancy (15.9 ± 0.3%) yet exhibited strongly diminished activities (e.g., EV^2+^‐dependent formate oxidation: 4.95 ± 0.26 U·mg^−1^ versus 70.90 ± 2.07 U·mg^−1^ for wild type) (Table S9). Thus, substantial activity loss can occur even when FMN remains associated, indicating that the aromatic network surrounding the FMN pocket is required for productive EV‐dependent turnover beyond simply maintaining FMN binding. Introduction of K237A into this background eliminated detectable FMN (N.D.) and further reduced activity (Table S9), supporting that FMN loss and aromatic network disruption contribute additively to impairment.

To further evaluate how the introduced mutations affect the interaction of ethyl viologen with *Me*FDH1, docking simulations were performed, and the calculated interaction energies are summarized in Table S10. The wild‐type enzyme exhibited a moderately favorable predicted interaction energy for EV (−27.40 kcal·mol^−1^). Removal of FMN through the K237A and K237E substitutions resulted in less favorable interaction energies (−20.81 and −22.44 kcal·mol^−1^, respectively), consistent with the reduced EV‐dependent catalytic activities observed experimentally. Introduction of additional aromatic‐residue substitutions led to non‐linear changes in the calculated interaction energies. Notably, several variants (including K237A/F471A and K237A/Y329A) displayed more negative interaction energies (−35.04 and −35.80 kcal·mol^−1^, respectively), reflecting alternative EV interaction poses stabilized by remaining aromatic residues. However, despite these favorably predicted interaction energies, these variants exhibited substantially reduced catalytic efficiencies (Table 1), indicating that strong local EV interactions alone are insufficient for efficient turnover. Similarly, the quadruple mutant K237A/F232A/F471A/Y329A was predicted to have the most favorable interaction energy (−37.75 kcal·mol^−1^), yet catalytic activity was nearly abolished. These observations are consistent with a common phenomenon in enzymology in which tighter ligand association can become nonproductive—for example, by stabilizing an orientation that is poorly coupled for electron transfer or by slowing mediator exchange—thereby decoupling apparent binding strength from catalytic output [52]. Accordingly, productive EV‐mediated catalysis likely requires not only energetic stabilization of the mediator in the FMN pocket region, but also a properly organized electron‐transfer pathway that supports rapid electron exchange with the β‐subunit redox relay (including B1), within the distance regime compatible with biological electron tunneling [49].

## Conclusion

In this study, we elucidated the functional interaction site and modes of the artificial electron mediator ethyl viologen (EV) in the tungsten‐containing formate dehydrogenase 1 from *Methylorubrum extorquens* AM1 (*Me*FDH1). By selectively disrupting the native cofactor‐interaction environment through the removal of FMN, we demonstrated that although FMN functions as the primary electron‐transfer hub and engages in direct aromatic interactions with EV, it is not the sole determinant of EV‐dependent catalysis. The unexpected retention of substantial formate oxidation and CO_2_ reduction activities in FMN‐deficient variants revealed the presence of an alternative, FMN‐independent interaction mode.

Through a combination of site‐directed mutagenesis, steady‐state kinetic analysis, molecular docking simulations, and structural integrity assessment, we identified residues Y329, F471, and F232 as key components of this secondary ethyl viologen interaction region. These aromatic residues act cooperatively to stabilize EV through π–π interactions and to position the mediator within electron‐transfer distance of the proximal B1 Fe‐S cluster. The progressive decrease in catalytic efficiency (*k*_cat_/*K*_M_) observed for the double and triple mutants, together with the near‐complete loss of activity in the quadruple mutant (K237A/F232A/F471A/Y329A) at low EV concentrations, underscores the functional importance of this aromatic network for productive mediator recruitment and orientation in the absence of the natural cofactor. Circular dichroism analysis further confirmed that these functional impairments arise from localized disruption of electron‐transfer interactions rather than from global structural destabilization of the enzyme.

Collectively, these findings advance our understanding of how artificial redox mediators interact with complex metalloenzymes and demonstrate that *Me*FDH1 employs a dual interaction strategy for EV‐mediated electron transfer. This insight provides a framework for the rational engineering of *Me*FDH1 variants with improved compatibility toward artificial mediators. Optimization of the spatial arrangement and electronic properties of mediator‐interacting residues may enable enhanced catalytic performance while reducing the concentrations of costly or cytotoxic mediators required. Ultimately, this work supports the development of efficient and robust biocatalytic systems for CO_2_ conversion under mild and industrially relevant conditions.

## Author contributions

EGP planned the experiments, performed bioinformatic analyses and gene cloning, and carried out the biochemical characterization and kinetic analyses. EGP also conducted feasibility evaluations. All experimental work was performed under the supervision of YHK. EGP drafted the original manuscript. YHK and EGP reviewed and edited the manuscript.

## Supporting information


**Table S1.** Plasmids and primers used for site‐directed mutagenesis of *Me*FDH1.
**Table S2.** FMN occupancy of *Me*FDH1 across independent purifications performed under anaerobic conditions.
**Table S3.** FMN add‐back test using FPLC‐purified *Me*FDH1 under anaerobic and dark conditions: FMN occupancy and EV/NAD‐dependent activities.
**Table S4.** Reproducibility of *Me*FDH1‐specific activities across independent Ni–NTA purifications under anaerobic conditions.
**Table S5.** Alanine‐scanning screen of aromatic residues surrounding the FMN pocket in *Me*FDH1 (Ni–NTA preparations).
**Table S6.** Specific activities (U·mg^−1^) of wild‐type *Me*FDH1 and mutant variants in formate oxidation and CO_2_ reduction using NAD^+^ or ethyl viologen (EV) as electron mediators.
**Table S7.** Specific activities (U·mg^−1^) of wild‐type *Me*FDH1 and mutants using different concentrations of ethyl viologen (EV) as the electron mediator in formate oxidation reaction.
**Table S8.** Estimated secondary structure composition of wild‐type *Me*FDH1 and mutant variants derived from far‐UV circular dichroism spectra using the BeStSel algorithm.
**Table S9.** Comparison of FMN occupancy, specific activities, and EV kinetic parameters for the aromatic triple mutant with and without the FMN‐disrupting K237A substitution.
**Table S10.** Calculated binding and interaction energies for ethyl viologen (EV) with wild‐type *Me*FDH1 and mutant variants.
**Fig. S1.** Anaerobic experimental setup.
**Fig. S2.** Docking pose of ethyl viologen (EV) in the K237E *Me*FDH1 mutant.
**Fig. S3.** Circular dichroism (CD) spectra of wild‐type *Me*FDH1 and mutant variants.

## Data Availability

All data are available in the main text and the Supporting Information. The datasets generated and analyzed during the current study are available from the corresponding author upon reasonable request.
