# Supplementary material for: An unexpected alternative viologen electron mediator site in tungsten‐containing formate dehydrogenase
Source: FEBS Lett. 2026 May 28;600(15):2094–107. doi: 10.1002/1873-3468.70373 (PMC13456608; doi:10.1002/1873-3468.70373)
Supplement: Supplementary file 1 [file FEB2-600-2094-s001.docx]

Supporting Information

**An unexpected alternative viologen electron mediator site in tungsten-containing formate dehydrogenase**

Eleni G. Poloniataki^1^, and Yong Hwan Kim^1,2^*

^1^School of Energy and Chemical Engineering, Ulsan National Institute of Science and Technology (UNIST), 50 UNIST-gil, Ulsan 44919, Republic of Korea

^2^Graduate School of Carbon Neutrality, Ulsan National Institute of Science and Technology (UNIST), 50 UNIST-gil, Ulsan 44919, Republic of Korea

*Corresponding authors:

Yong Hwan Kim, School of Energy and Chemical Engineering and Graduate School of Carbon Neutrality, Ulsan National Institute of Science and Technology (UNIST), 50 UNIST-gil, Ulsan 44919, Republic of Korea

telephone number: +82-52-217-3068, e-mail: [metalkim@unist.ac.kr](mailto:metalkim@unist.ac.kr)

### **Table S1. Plasmids and primers used for site-directed mutagenesis of *Me*FDH1.**

The parental plasmid pCM110–FDH1 was used to generate single, double, triple, and quadruple mutants by site-directed mutagenesis. Primer names and nucleotide sequences are listed in the 5′–3′ direction. Mutations were introduced sequentially using the indicated plasmid templates.

| **Mutation** | **Plasmid** | **Primer** | **Primer’s Sequence (5’ to 3’)** |
| --- | --- | --- | --- |
| Wildtype | pCM110-FDH1 | - | - |
| K237A | pCM110-FDH1 | Beta-K237A-F | cccaccggccgcgcctggcgctccgtgcgcggcgagcc |
|  |  | Beta-K237A-R | cacggagcgccaggcgcggccggtgggaaagcc |
| K237E | pCM110-FDH1 | Beta-K237E-F | cccaccggccgcgagtggcgctccgtgcgcggcg |
|  |  | Beta-K237E-R | cacggagcgccactcgcggccggtgggaaagcc |
| K237A/F232A | pCM110-FDH1(βK237A) | Beta-F232A-F | ggcggcgccggcgcccccaccggccgcaagtgg |
|  |  | Beta-F232A-R | gcggccggtgggggcgccggcgccgccgaggcc |
| K237A/F471A | pCM110-FDH1(βK237A) | Beta-F471A-F | aaatacggctgcgccatcggctcggccgcgg |
|  |  | Beta-F471A-R | ccgagccgatggcgcagccgtatttttccag |
| K237A/Y329A | pCM110-FDH1(βK237A) | Beta-Y329A-F | ggggccggcgccgccatctgcggcgaggaatc |
|  |  | Beta-Y329A-R | tcgccgcagatggcggcgccggccccacggcg |
| K237A/F471A/Y329A | pCM110-FDH1 (βK237A/Y329A) | Beta-F471A-F | aaatacggctgcgccatcggctcggccgcgg |
|  |  | Beta-F471A-R | ccgagccgatggcgcagccgtatttttccag |
| K237A/F232A/F471A/Y329A | pCM110-FDH1 (βK237A/F471A/Y329A) | Beta-F232A-F | ggcggcgccggcgcccccaccggccgcaagtgg |
|  |  | Beta-F232A-R | gcggccggtgggggcgccggcgccgccgaggcc |

**Table S2. FMN occupancy of *Me*FDH1 across independent purifications performed under anaerobic conditions.**
FMN occupancy (%) was measured for *Me*FDH1 collected (i) directly after Ni–NTA elution and (ii) after the same Ni–NTA eluate was further purified by FPLC (polishing step), to assess how much FMN remains associated with the enzyme after additional purification. FMN occupancy (%) was determined for *Me*FDH1 samples collected after the Ni–NTA elution step and after FPLC polishing. Occupancy was calculated as (mol FMN/mol *Me*FDH1)×100. Values for Purifications 1–3 represent independent purification batches; **each purification sample was measured three times,** and the within-purification values are reported as mean ± SD. The final row reports the mean ± SD **between** independent purifications. All purification steps were carried out inside an anaerobic chamber to minimize cofactor loss and oxidative degradation.

| **Purification** | **Ni-NTA**  **(FMN occupancy, %)** | **FPLC**  **(FMN occupancy, %)** |
| --- | --- | --- |
| 1 | 20.8 ± 0.7 | 5.5 ± 0.07 |
| 2 | 33.2 ± 1.4 | 8.2 ± 1.0 |
| 3 | 17.2 ± 0.1 | 6.5 ± 0.2 |
| Mean ± SD (between purifications) | 23.7 ± 8.4 | 6.7 ± 1.4 |

**Table S3. FMN add-back test using FPLC-purified *Me*FDH1 under anaerobic and dark conditions: FMN occupancy and EV/NAD-dependent activities.**
*Me*FDH1 was purified by Ni–NTA and further polished by FPLC; the resulting FPLC fraction was used for FMN add-back experiments. Exogenous FMN was added at the indicated molar equivalents relative to *Me*FDH1 (**×1 FMN denotes a 1:1 molar ratio, i.e., mol(*Me*FDH1) = mol(FMN) added**). Samples were incubated at room temperature for 2 h **inside an anaerobic chamber and protected from light.** After incubation, **free FMN was removed**, and FMN concentration was quantified from the recovered **protein fraction**. FMN occupancy was calculated as (mol FMN/mol *Me*FDH1)×100. Specific activities were then measured for formate oxidation with NAD^+^, formate oxidation with oxidized ethyl viologen (EV^2+^), and CO_2_ reduction with reduced ethyl viologen radical (EV•^+^). Activities are reported as U·mg^-1^ (µmol·min^-1^·mg^-1^). Values are mean ± SD (n = 3).

| **Condition**  **(FMN added)** | **FMN added (equiv.)** | **Measured FMN concentration (μΜ)** | **FMN occupancy (%)** | **Formate oxidation (NAD^+^) (U·mg^-1^)** | **Formate oxidation (EV)**  **(U·mg^-1^)** | **CO_2_ Reduction (EV) (U·mg^-1^)** |
| --- | --- | --- | --- | --- | --- | --- |
| Without adding FMN | - | 0.13 ± 0.02 | 8.2 ± 1.0 | 29.78 ± 2.64 | 95.20 ± 4.92 | 62.05 ± 7.68 |
| x1 FMN | 1 | 0.15 ± 0.003 | 7.3 ± 0.1 | 27.04 ± 3.61 | 63.02 ± 2.43 | 64.83 ± 6.79 |
| x2 FMN | 2 | 0.19 ± 0.009 | 9.3 ± 0.5 | 32.02 ± 1.85 | 90.46 ± 7.08 | 64.44 ± 3.75 |
| x5 FMN | 5 | 0.20 ± 0.003 | 10.2 ± 0.1 | 32.05 ± 1.84 | 90.54 ± 7.08 | 64.50 ± 3.75 |
| x10 FMN | 10 | 0.19 ± 0.006 | 9.6 ± 0.3 | 35.06 ± 0.13 | 75.59 ± 0.91 | 56.04 ± 4.29 |

**Table S4. Reproducibility of *Me*FDH1 specific activities across independent Ni–NTA purifications under anaerobic conditions.**
Specific activities (U·mg^-1^; µmol·min^-1^·mg^-1^) for formate oxidation with NAD^+^, formate oxidation with oxidized ethyl viologen (EV^2+^), and CO_2_ reduction with reduced ethyl viologen radical (EV^•+^) were measured using **Ni–NTA eluates only**. The samples reported in this table were obtained from **independent Ni–NTA purifications** and are **distinct from the preparations used for Tables S2 and S3**. Each purification sample was measured three times and reported as mean ± SD. The final row reports the mean ± SD **between** independent purifications. All purification and activity measurements were carried out under anaerobic conditions.

| **Purification** | **Formate oxidation (NAD^+^)**  **(U·mg^-1^)** | **Formate oxidation (EV)**  **(U·mg^-1^)** | **CO_2_ Reduction (EV)**  **(U·mg^-1^)** |
| --- | --- | --- | --- |
| 1 | 29.67 ± 0.12 | 64.13 ± 2.81 | 42.52 ± 4.48 |
| 2 | 38.96 ± 2.36 | 71.47 ± 2.57 | 32.04 ± 4.02 |
| 3 | 32.01 ± 0.14 | 68.45 ± 4.52 | 41.57 ± 1.92 |
| Mean ± SD (between purifications) | 33.55 ± 4.83 | 68.02 ± 3.69 | 38.71 ± 5.80 |

**Table S5. Alanine-scanning screen of aromatic residues surrounding the FMN pocket in *Me*FDH1 (Ni–NTA preparations).**
To identify residues that influence EV-dependent turnover, alanine scanning mutagenesis was performed for selected aromatic residues located within ~10 Å of the FMN cofactor in the β-subunit. Specific activities (U·mg^-1^; µmol·min^-1^·mg^-1^) were measured for formate oxidation with NAD^+^, formate oxidation with oxidized ethyl viologen (EV^2+^), and CO_2_ reduction with reduced ethyl viologen radical (EV^•+^) under the standard assay conditions described in Methods. All proteins were purified by Ni–NTA and assayed under anaerobic conditions. Each activity value represents the mean ± SD of three measurements (n = 3). This screening dataset was used to prioritize residues for further mechanistic analysis in combination with the K237A background to eliminate FMN binding.

| **Mutation** | **Formate oxidation (NAD^+^)**  **(U·mg^-1^)** | **Formate oxidation (EV)**  **(U·mg^-1^)** | **CO_2_ Reduction (EV)**  **(U·mg^-1^)** |
| --- | --- | --- | --- |
| Wildtype | 32.07 ± 0.23 | 70.90 ± 2.07 | 41.56 ± 1.92 |
| F232A | 0.6 ± 0.46 | 40.04 ± 2.48 | 16.72 ± 1.55 |
| F354A | 34.49 ± 2.19 | 72.82 ± 6.19 | 44.18 ± 2.62 |
| F356A | 10.39 ± 0.42 | 64.39 ± 7.79 | 32.20 ± 9.98 |
| F462A | 0.009 ± 0.006 | 72.01 ± 1.66 | 39.73 ± 0.59 |
| F471A | 15.90 ± 2.55 | 43.34 ±0.89 | 15.32 ± 3.85 |
| Y329A | 0.62 ± 0.09 | 25.41 ± 1.96 | 11.18 ± 1.64 |

**Table S6. Specific activities (U·mg⁻¹) of wild-type *Me*FDH1 and mutant variants in formate oxidation and CO_2_ reduction using NAD^+^ or ethyl viologen (EV) as electron mediators.**

Specific activities for formate oxidation with NAD⁺, formate oxidation with oxidized EV, and CO_2_ reduction with reduced EV were measured for wild-type *Me*FDH1 and single, double, triple, and quadruple mutants. Values represent average ± standard deviations from triplicate measurements.

| **Mutation** | **Formate oxidation (NAD^+^)**  **(U·mg^-1^)** | **Formate oxidation (EV)**  **(U·mg^-1^)** | **CO_2_ Reduction (EV)**  **(U·mg^-1^)** |
| --- | --- | --- | --- |
| Wildtype | 32.07 ± 0.23 | 70.90 ± 2.07 | 41.56 ± 1.92 |
| K237A | 0.005 ± 0.006 | 21.86 ± 0.48 | 11.00 ± 0.20 |
| K237E | 0.008 ± 0.008 | 20.28 ± 0.81 | 10.91 ± 0.30 |
| K237A/F232A | 0 ± 0 | 15.36 ± 1.01 | 8.78 ± 0.76 |
| K237A/F471A | 0 ± 0 | 11.04 ± 1.62 | 6.29 ± 1.47 |
| K237A/Y329A | 0.005 ± 0.003 | 12.86 ± 1.11 | 6.42 ± 0.08 |
| K237A/F471A/Y329A | 0 ± 0 | 4.95 ± 0.39 | 3.10 ± 0.31 |
| K237A/F232A/F471A/Y329A | 0.008 ± 0.012 | 3.65 ± 0.20 | 2.78 ± 0.34 |

**Table S7. Specific activities (U·mg⁻¹) of wild-type *Me*FDH1 and mutants using different concentrations of ethyl viologen (EV) as the electron mediator in formate oxidation reaction.**

Specific activities were measured at 10 mM, 1.25 mM, and 0.16 mM EV to evaluate mediator-dependent performance across wild-type *Me*FDH1 and single, double, triple, and quadruple mutants. Values represent average ± standard deviations from triplicate experiments.

| **Mutation** | **10 mM EV** | **1.25 mM EV** | **0.16 mM EV** |
| --- | --- | --- | --- |
| Wildtype | 70.90 ± 2.07 | 34.00 ± 1.64 | 11.48 ± 1.26 |
| K237A | 21.86 ± 0.48 | 7.07 ± 0.43 | 0.32 ± 0.06 |
| K237E | 20.28 ± 0.81 | 7.53 ± 0.25 | 1.52 ± 0.13 |
| K237A/F232A | 15.36 ± 1.01 | 7.49 ± 0.21 | 2.26 ± 0.09 |
| K237A/F471A | 11.04 ± 1.62 | 4.37 ± 0.25 | 0.32 ± 0.06 |
| K237A/Y329A | 12.86 ± 1.11 | 2.99 ± 0.37 | 0.53 ± 0.03 |
| K237A/F471A/Y329A | 4.95 ± 0.39 | 1.46 ± 0.11 | 0 ± 0 |
| K237A/F232A/F471A/Y329A | 3.65 ± 0.20 | 1.26 ± 0.05 | 0.01 ± 0.02 |

**Table S8. Estimated secondary structure composition of wild-type *Me*FDH1 and mutant variants derived from far-UV circular dichroism spectra using the BeStSel algorithm.**

Secondary structure contents (α-helix, antiparallel and parallel β-sheet, turns, and unordered regions) were calculated from CD spectra (190–260 nm) using the BeStSel method. Values are reported as percentages (%) of total secondary structure content. The analysis indicates that the overall secondary structure distribution is largely preserved across wild-type *Me*FDH1 and all mutants, suggesting that the introduced mutations do not cause major global folding disruptions.

| **Estimated secondary structure content (%)** | | | | | | | | | | | | | | | | | |
| --- | --- | --- | --- | --- | --- | --- | --- | --- | --- | --- | --- | --- | --- | --- | --- | --- | --- |
| **Secondary structure** | | **Wildtype** | | **K237A** | | **K237E** | | **K237A/F232A** | | **K237A/F471A** | | **K237A/Y329A** | | **K237A/F471A/ Y329A** | | **K237A/F232A/ F471A/Y329A** | |
| Helix | Helix1 (regular) | 42.6 | 26.8 | 32.5 | 17.6 | 35.9 | 20.9 | 57.2 | 39.4 | 48.1 | 30.9 | 36.2 | 22.0 | 46.5 | 29.3 | 48.5 | 30.3 |
|  | Helix2 (distorted) |  | 15.8 |  | 14.9 |  | 15.0 |  | 17.8 |  | 17.3 |  | 14.2 |  | 17.1 |  | 18.2 |
| Antiparallel | Ant1 (left-twisted) | 1.6 | 0.0 | 3.4 | 0.0 | 1.6 | 0.0 | 4.6 | 0.0 | 2.3 | 0.0 | 6.0 | 0.0 | 2.0 | 0.0 | 0.2 | 0.0 |
|  | Anti2 (relaxed) |  | 0.0 |  | 3.0 |  | 1.1 |  | 0.0 |  | 0.0 |  | 6.0 |  | 0.0 |  | 0.0 |
|  | Anti3 (right-twisted) |  | 1.6 |  | 0.4 |  | 0.4 |  | 4.6 |  | 2.3 |  | 0.0 |  | 2.0 |  | 0.2 |
| Parallel | | 2.0 | | 6.9 | | 6.5 | | 0.0 | | 0.6 | | 6.9 | | 0.4 | | 1.9 | |
| Turn | | 11.4 | | 12.0 | | 12.0 | | 5.1 | | 8.5 | | 11.5 | | 7.5 | | 7.9 | |
| Others | | 42.4 | | 45.1 | | 44.0 | | 33.0 | | 40.5 | | 39.4 | | 43.6 | | 41.5 | |

**Table S9. Comparison of FMN occupancy, specific activities, and EV kinetic parameters for the aromatic triple mutant with and without the FMN-disrupting K237A substitution.**
Wild-type *Me*FDH1, the aromatic triple mutant (F232A/F471A/Y329A), and the corresponding quadruple mutant in the K237A background (K237A/F232A/F471A/Y329A) were purified under the same conditions. FMN occupancy was quantified as (mol FMN/mol *Me*FDH1)×100; “N.D.” indicates FMN was not detected. Specific activities (U·mg^-1^; µmol·min^-1^·mg^-1^) were measured for NAD^+^-dependent formate oxidation, EV^2+^-dependent formate oxidation, and EV^•+^-dependent CO_2_ reduction. Michaelis–Menten parameters (*K_M_*, *k_cat_* and *k_cat_*/*K_M_*) were obtained from EV^2+^ concentration series as described in Methods. Values are mean ± SD (n = 3).

| **Mutation** | **FMN occupancy (%)** | **Formate oxidation (NAD^+^) (U·mg^-1^)** | **Formate oxidation (EV)**  **(U·mg^-1^)** | **CO_2_ Reduction (EV) (U·mg^-1^)** | ***K_M_***  **(mM)** | ***k_cat_***  **(s^-1^)** | ***k_cat_/K_M_***  **(s^-1^·mM^-1^)** |
| --- | --- | --- | --- | --- | --- | --- | --- |
| Wildtype | 19.9 ± 0.9 | 32.07 ± 0.23 | 70.90 ± 2.07 | 41.56 ± 1.92 | 0.91 ± 0.004 | 715.03 ± 17.69 | 785.76 ± 19.74 |
| F232A/F471A/Y329A | 15.9 ± 0.3 | 0.02 ± 0.003 | 4.95 ± 0.26 | 3.02 ± 0.22 | 2.54 ± 0.007 | 179.45 ± 13.12 | 70.65 ± 5.17 |
| **K237A**/F232A/F471A/Y329A | N.D | 0.008 ± 0.012 | 3.65 ± 0.20 | 2.78 ± 0.34 | 3.13 ± 0.009 | 69.01 ± 5.68 | 22.05 ± 1.82 |

**Table S10. Calculated binding and interaction energies for ethyl viologen (EV) with wild-type *Me*FDH1 and mutant variants.**

Binding energy, ligand energy, protein energy, complex energy, and entropic energy were obtained from docking simulations to compare the interaction strength of EV with the wild-type enzyme and with single, double, triple, and quadruple mutants. More negative binding energy values indicate stronger predicted EV–protein interactions. Docking simulations were performed using CB-Dock2, and energies were calculated using Discovery Studio.

| **Mutation** | **Binding Energy (kcal/mol)** | **Ligand Energy (kcal/mol)** | **Protein Energy (kcal/mol)** | **Complex Energy (kcal/mol)** | **Entropic Energy (kcal/mol)** |
| --- | --- | --- | --- | --- | --- |
| Wildtype | -27.40 | 156.85 | -61410.64 | -61281.19 | 18.52 |
| K237A | -20.81 | 163.29 | -60855.28 | -60712.79 | 18.51 |
| K237E | -22.44 | 172.66 | -60887.11 | -60736.90 | 18.51 |
| K237A/F232A | -22.86 | 164.27 | -60842.64 | -60701.24 | 18.51 |
| K237A/F471A | -35.04 | 157.35 | -60918.85 | -60796.54 | 18.53 |
| K237A/Y329A | -35.80 | 166.38 | -60933.15 | -60802.57 | 18.53 |
| K237A/F471A/Y329A | -28.68 | 166.93 | -60926.73 | -60788.48 | 18.53 |
| K237A/F232A/F471A/Y329A | -37.75 | 172.25 | -60298.16 | -60163.66 | 18.53 |

**Figure S1. Anaerobic experimental setup.** Photographs of the anaerobic chamber workstations (Coy Laboratory Products) used in this study: (A) sonication chamber for cell lysis, (B) UV/Vis chamber for activity measurements, (C) FPLC chamber for purification and (D) CAM-12 H_2_/O_2_ detector. All chambers were operated under an N_2_/H_2_ atmosphere and equipped with CAM-12 H_2_/O_2_ detectors.

**Figure S2. Docking pose of ethyl viologen (EV) in the K237E *Me*FDH1 mutant.**

Docking simulation of the mutant showing the predicted orientation of ethyl viologen (EV) relative to the surrounding aromatic residues F232, F471, and Y329, as well as the K237E substitution in the absence of FMN. The K237E mutation leads to FMN dissociation from *Me*FDH1, thereby disrupting the π–π interactions with F471, as present in the wild type, and decreasing the EV–Y329 distance to 4.5 Å.

**Figure S3. Circular dichroism (CD) spectra of wild-type *Me*FDH1 and mutant variants.**

Far-UV CD spectra (190–260 nm) of wild-type *Me*FDH1 and the indicated single, double, and multiple mutants were recorded to assess their secondary-structure content. All variants display similar spectral profiles characterized by minima near ~208 and ~222 nm, indicating that the overall secondary structure is largely preserved upon mutation.
